# Developing Decision-Making Expertise in Professional Sports Staff: What We Can Learn from the Good Judgement Project

**DOI:** 10.1186/s40798-023-00629-w

**Published:** 2023-10-25

**Authors:** P. J. Wilson, John Kiely

**Affiliations:** 1 Setanta College, Limerick, Ireland; 2 Department of PE and Sports Sciences, University of Limerick, Limerick, V94 T9PX Ireland

**Keywords:** Professional sport, Decision-making, Forecasting, Judgement, Uncertainty, Intuition, Experience, Expertise

## Abstract

Success within performance sports is heavily dependent upon the quality of the decisions taken by educated and experienced staff. Multi-disciplinary teams (MDTs) typically collate voluminous data, and staff typically undergo extensive and rigorous technical and domain-specific training. Although sports professionals operate in sometimes volatile, uncertain, complex and ambiguous decision-making environments, a common assumption seems to be that education and experience will automatically lead to enhanced and effective decision-making capabilities. Accordingly, there are few formal curriculums, in coaching or sports science contexts, focussed on translating the extensive research on judgement and decision-making expertise to professional sports staff. This article aims to draw on key research findings to offer insights and practical recommendations to support staff working within professional performance contexts. Through this distillation, we hope to enhance understanding of the factors underpinning effective decision-making in dynamic, high-stakes professional sporting environments. Broadly, the conclusions of this research demonstrate that decision-making efficacy is enhanced through application of three specific strategies: (i) Design of more engaging professional cultures harnessing the power of collectives encouraging diverse opinions and perspectives, and fostering and promoting collaborative teamwork, (ii) education specifically targeting debiasing training, designed to counter the most common cognitive pitfalls and biases and, (iii) the implementation of evaluation strategies integrating rigorous testing and real-time feedback.

## Key points

- Forecasting, judgement and decision-making tasks, in professional sports, typically occur in volatile, uncertain, complex and ambiguous contexts
- Effective forecasting, judgement and decision-making skills are not the inevitable outcomes of experience alone
- Forecasting, judgement and decision-making capacities are trainable skills enhanced by focused education and appropriately engineered decision-making processes

## Introduction

Inspired by the failure of intelligence analysts to predict significant socio-political events (most notably missing warning signs of 9/11), the United States government responded by creating the Intelligence Advanced Research Projects Activity (IARPA) [1]. IARPA’s objective was to engage in innovative, high-payoff research designed to tackle the most difficult challenges facing the intelligence community (IC). A core strand of this research focussed on developing insights into the mutually entwined topics of forecasting and decision-making [2].

By 2010, IARPA had successfully implemented classified prediction markets—forums where participants trade contracts whose payoffs depend on successfully forecasting clearly defined future outcomes. Here, IC employees, with access to top-secret information, could ‘bet’ on future forecasts. Inspired by early successes of these closed forecasting markets, IARPA speculated that *wisdom of the crowd*—the aggregation of multiple, independent judgements—could be leveraged to further enhance predictive accuracy [3]. And so, in 2011, IARPA launched a public forecasting tournament.

Over the following 4 years, five university-based research teams were invited to answer hundreds of real-world questions suffused with ambiguity, uncertainty, time-pressure and incomplete information (such as, *Will North Korea launch a multi-stage missile before May 10th, 2014?*). Teams failing to meet consistent standards were eliminated. After 2 years, a single team remained. This team, labelled *Good Judgement,* consisted of amateur forecasters assembled by Wharton professors Philip Tetlock and Barbara Mellers. The *Good Judgement* team demonstrated an astonishing dominance, outperforming individual forecasters by 60%, academic teams by 40% and regularly outperformed intelligence analysts with privileged access to classified information [4]. Notably, *Good Judgement* showed no regression toward the mean as the tournament progressed. Instead, all psychometric indices of judgmental accuracy—such as calibration, discrimination and area under the curve—improved across time [5].

Previously, in a 20-year study evaluating nearly 30,000 predictions from 284 subject-experts, Tetlock had assessed the accuracy of experts’ probability judgments across a diversity of topics [6]. The results highlighted the limitations of human forecasting abilities, specifically illustrating that overconfidence, hindsight bias and self-serving counterfactual reasoning were pervasive amongst experienced experts [6, 7]. Tetlock’s data demonstrated that experts typically over-inflated both their forecasting abilities and the accuracy of their predictions. Yet, paradoxically, experts’ predictions commonly failed to outperform even simple statistical models (such as randomly generated guesses). As Tetlock noted, experts judgements were about as accurate as "a dart-throwing chimpanzee" [6].

Within professional sports contexts, we typically assume that experience and a track-record of prior competitive successes validates good forecasting and decision-making abilities. This presumption, however, conflicts with data collated across a multitude of complex human endeavours. Evidence illustrates that experience alone is an inefficient and ineffective means of reliably enhancing decision-making capabilities. In exploring the potential relevance of decision-making science to professional sports domains, here, we review and extract evidence-led and actionable learning points which, if implemented, could potentially inform and enhance decision-making cultures within professional sporting contexts.

## Why is Professional Sport Vulnerable to Poor Forecasting and Decision-Making?

Success, within performance sports, is heavily dependent upon the quality of the decisions taken by professional staff-coaching, conditioning, rehabilitation and medical—who, collectively, form the multi-disciplinary teams that direct, nurture, and advise performers [8]. Sports science support teams, although frequently supported by sophisticated technologies, must nevertheless rely on individual, and combined, intellects and experiences to accurately forecast likely future outcomes and to subsequently decide on courses of action optimally aligned to organisational objectives. Multi-disciplinary support team members are typically highly trained with extensive practical experience, with specific tertiary education and specialist qualifications commonplace. Such staff are rightly considered domain-specific experts, having both training and experience in specific facets of high-performance sport [8]. Clubs, governing bodies and sporting organisations endeavour to recruit the best available personnel in the belief that, both collectively and individually, their expertise and experience will enable them to make ‘good’ decisions. Sports professionals regularly make time-pressured, high-stakes decisions under conditions of uncertainty and incomplete information [9].

While technologies, data analysis methodologies and staff educational standards have all dramatically improved in recent decades [10, 11], whether this has substantially improved the quality of decision-making remains unclear. This suggestion may seem counter-intuitive, but consider the problems currently being experienced within medical domains. In recent years, a number of publications in the world’s premier medical journals have launched dedicated initiatives focussed on educating medical practitioners on the damaging decision-making consequences of too many metrics and too much data [12–15]. Conventional wisdom assumes more information always drives better decision-making. Yet the excessive screening, scanning and testing pervasive across numerous medical disciplines are collectively driving, what has recently been described as, a ‘Tsunami of overtreatment’; an epidemic of over-testing, overdiagnosis and over-medicalisation [16]. The problems caused by these linked phenomena are far from trivial and are acknowledged as one of medicine’s most damaging and costly problems, to the extent that overdiagnosis has recently been labelled, in the *British Medical Journal,* as “the most important story in contemporary healthcare” [16, 17].

As illustrated by the problems associated with overdiagnosis, more data does not automatically drive better decision-making. Critically, the influences confounding and complicating effective decision-making in medical contexts, such as information overload and measurement blindness, are also evident within high-performance sporting environments [16]. Within professional sporting contexts, there is a similar proliferation of too much undifferentiated, unweighted, non-scientifically supported, irrelevant and non-credible data being collected alongside, and weighted equivalently to, relevant, empirically justified, and credible data.

Notably, previous work has advocated the benefits of embedding research and development processes into the daily work of professional sports practitioners (for example, see McCall 2016 [18]). Such initiatives sensibly offer real value by enhancing decision-making outcomes within performance contexts. The perspectives offered here are not an argument against data-informed decision-making processes, yet do serve to underline the problems inherent in assuming that more data automatically and directly drives better decision-making outcomes. Within sporting contexts, the outcomes of any given intervention—whether tactical, training-related, or therapeutic—are inherently uncertain and decision-making tasks are complicated by a diversity of interacting, conspiring influences such as poor data management, incorrect modelling assumptions, high sensitivity of estimates, lack of epidemiological features, poor evidence of the efficacy of conventional interventions, lack of transparency, logical errors, lack of determinacy and, notably, strong ideological presumptions [19, 20].

Decision-making within fast-moving, high-pressure sporting contexts is inherently difficult. The drivers of this decision-making difficulty, and the potential remedies serving to enhance decision-making efficacy, however, are not always readily apparent.

### Decision-Making in VUCA Environments

**Fig. 1:**
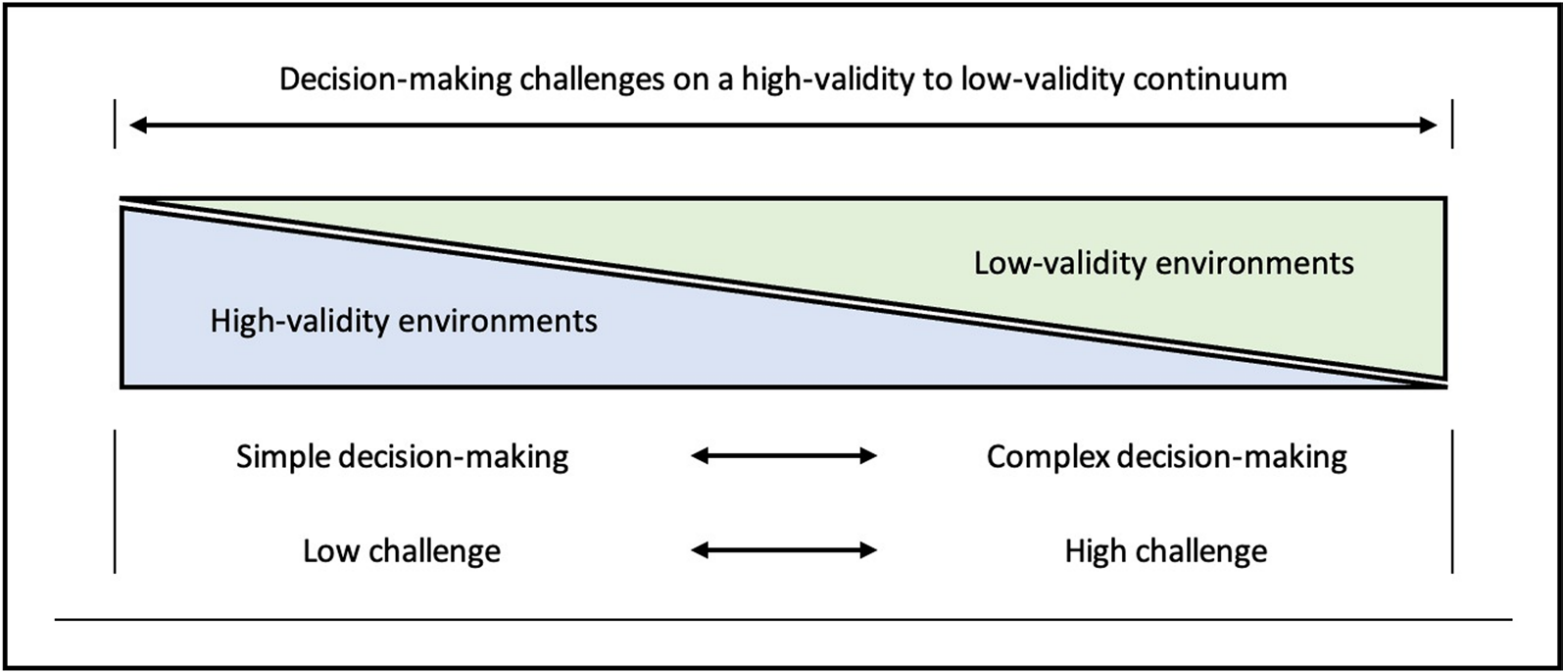
The high-to-low validity decision-making continuum

Certain environments are highly susceptible to forecasting and decision-making errors. Such environments are typically characterised as volatile, uncertain, complex and ambiguous and often described using the acronym VUCA [21–24]. In these environments, outcomes are highly unpredictable and predictions, accordingly, have low to zero validity [25]. Practitioners tasked with making decisions in low validity contexts cannot rely on intuition-informed guidance. Why? Because in zero validity environments there are no coherent, identifiable, ‘learnable’ patterns. Repeat exposure to such phenomena does not reveal the reliable cues and clues necessary to bestow foresight. Phenomena in high validity environments, in contrast, do reveal consistent patterns of behaviour; patterns that can be learned through repeat exposure. The development and refinement of skilled intuitions require high-validity inputs [25]. Context-specific dimensions of professional sports practice, accordingly, can be conceptualised as existing on a zero-to-high validity continuum. In high-validity contexts, subsequently, the intuitions embedded via prior experience may provide meaningful decision-making guidance. In low-validity contexts, however, strong intuitions are likely to confound and impede effective decision-making (see Fig. 1).

VUCA environments are characterised by the complex interconnectedness of many interacting influences, all mutually co-modulating via densely entangled nonlinear feedforward and feedback information loops. Within VUCA environments, the direction and pace of change, subsequent to any interventions, are inherently unpredictable. Problems in VUCA environments are intractably entwined. Many potential solutions exist. There is no clear distinction between better and worse options. All solutions have knock-on consequences. All solutions offer a potential pay-off, yet all exert a cost. Similarly, each course of action presents a multitude of plausible counter-factual outcomes, each with similar probabilities.

Decision-making in VUCA domains is typically time-pressured, with inadequate and imperfect access to timely feedback and typically long lag times between introduced interventions and the (frequently vaguely assessed) realisation of desired outcomes [12–14]. Similarly, the problems posed in VUCA environments are inevitably novel and context-specific. Hence, little, or no, directly comparable historical data are available; conceptual understanding is incomplete; measurements are imprecise; evidence is imperfect and personal experiences are inevitably biased [22, 23, 26]. The perception that a particular solution worked previously, in an inevitably different context, does not suggest repeating that historical solution will work again. Accordingly, problems can be approached from multiple, sometimes competing, perspectives and have multiple possible solutions. The ‘best’ solution, however, cannot be determined in advance [26, 27].

In summary, the efficacy of decision-making in professional sports contests is likely impacted by the complexity and validity of the specific decision-making task. By recognising the nuances and inherent limitations of decision-making efficacy within different contexts, organisations and practitioners can more deliberately and strategically navigate the challenges inherent within the specific decision-making task.

### Is Service Provision in Professional Sport a VUCA Decision-Making Environment?

Within VUCA environments, human judgement and decision-making are inherently and irrevocably flawed. Clearly, within sporting contexts, some decision-making tasks are mundane, routine and hold very high validity. In such contexts, effective heuristics, or simple rules of thumb, provide fast, effective and accurate decision-making guides [28]. However, as complexity increases, and environmental validity decreases, intuition becomes less reliable [25].

Experienced practitioners, who intensively and critically reflect on and engage with their experiences, are more likely to develop the expertise to recognise high-complexity and low-validity decision-making contexts (see, for example, Ericsson 2008 [29]). In performance sports contexts, coaches, strength and conditioning, physiotherapists, medical and sports science staff are habitually faced with unique and complex problems permeated with differing predictive validities. The rehab specialist, for example, may be educated in the common injury mechanisms shared by a category of injury. Yet the personal characteristics of the injured player; the specific characteristics of the injury; the relationships, confidences and communications with key staff; the changing biological and psycho-emotional context within which the injury occurred and within which rehabilitation occurs; the available tools and technology; and practitioner biases ensure that every case is inevitably unique.

In practical contexts, the interpreted success of previous solutions to past problems is a powerful shaper of beliefs and biases. Yet, in high-challenge/low-validity decision-making contexts, an over-reliance on past decision-making successes is a fundamentally flawed, albeit intuitive, strategy. Accordingly, in complex environments, the inappropriate redeployment of past decision-making ‘solutions’, in subtly different contexts, is likely to diminish, rather than enhance, decision-making efficacy [25].

In summary, the high-stakes, impulsive, complex, and unstable nature of professional sport environments, in tandem with our innate cognitive limitations, conspire to severely constrain our capacity to compute and accurately forecast the future eventualities radiating from current decisions. Accordingly, experience-driven learning, in specific contexts, is inevitably biased, incomplete, unreliable, and fundamentally flawed. Importantly, recognising the implicit problems of decision-making within VUCA environments enables us to translate and apply decision-making guidelines established in VUCA contexts to professional sports domains.

## Learning from Adversarial Collaboration

The dominant conceptual framework, within the popular literature, remains the influential heuristics and biases paradigm formulated by Kahneman and Tversky [30], culminating in Kahneman being awarded the 2002 Nobel prize in Economics. Nevertheless, key aspects of this paradigm—specifically its diminishment of the value of intuition within complex decision-making processes—are not universally accepted. Most notably, the “Naturalistic Decision-Making” (NDM) framework, pioneered by psychologist Gary Klein, advocates that intuitive judgements, founded in prior experience, provide a more realistic reflection of how people make real-world decisions [31, 32]. Klein suggests intuitive judgements, what we colloquially call *gut feelings* or *gut instincts,* provide a proficient and reliable means of making ‘good’ decisions in specific contexts [31–33]. Klein frames *gut feelings* as potentially useful sources of information that’ should be carefully and deliberately evaluated, rather than casually dismissed or automatically acted upon.

NDM, accordingly, seeks to demystify intuition by identifying the cues that experts use to arrive at intuitive judgments, even if those cues involve tacit knowledge—insights derived from assimilated experiences—that are inherently difficult for the expert to recognise, articulate or systematise [31, 32]. Although the heuristics and biases approach has a healthy scepticism of the decision-making utility of intuition [25], the NDM approach promotes the value of expert intuition, of ‘*gut instinct’*, across a range of fields and professions.

Despite their philosophical differences, Kahneman and Klein collaborated to co-author a review—self-described as an *adversarial collaboration* [34, 35]—critically examining their compatible, and conflicting, perspectives [25]. In tackling the issue of *skilled intuition*, both endorsed a summary provided by Nobelist Herbert Simon [36]:“ *The situation has provided a cue: This cue has given the expert access to information stored in memory, and the information provides the answer. Intuition is nothing more and nothing less than recognition*” (p. 155).

### Developing Intuitive Expert Judgement

Kahneman and Klein suggested two essential conditions for the evolution of expert intuitive judgement [25]. Specifically, they observed that the quality of intuitive judgments is, firstly, dependent on the predictability of the environment in which the judgment is made and, secondly, the individual’s opportunity to learn from the regularities of that environment. To meaningfully learn from these regularities, practitioners require adequate experiential exposure and access to timely and reliable feedback. Accordingly, if there are recognisable cues within the environment that reliably enhance the predictability of subsequent events, and if the decision-maker has sufficient exposure to adequately discern the regularities of that environment, then intuitive judgements hold validity. In such contexts, pattern recognition informs judgements [25].

Kahneman and Klein [25] suggested that both medicine and firefighting are practiced in contexts within which intuitive judgments hold ‘fairly high validity’. In these environments, observable cues are of sufficiently high validity to provide meaningful indications of future outcomes. Additionally, practitioners are typically exposed to multiple opportunities to observe, and progressively learn from, these environmental cues. Such cues, even if not consciously recognised, positively influence judgement. Subsequently, through repeat exposure to relevant cues, the astute and discerning observer may gradually ‘learn’ patterns enabling them to take advantage of environmental regularities. Skilled intuitions, from this perspective, develop when behavioural patterns are of sufficient regularity to provide meaningfully informative cues directly enhancing the predictability of subsequent outcomes [25].

Activities such as stock market predictions or political forecasting, in contrast, do not generate consistent or reliable informational patterns and consequently are considered zero-validity environments [6]. Similarly, in sporting contexts, there is an evidenced inability of professional scouts to judge the potential of team sport players across a range of sports, despite extensive time-on-task and ample exposure to environmental cues [37]. Not all decision tasks are equal. Each exists on a continuum ranging from high to low validity (see Fig. 1). Importantly, previous experiences, in zero-validity contexts, are, from a judgement and decision-making perspective, worse than worthless, as they are potentially misleading.

### The Problem of Fractionated Expertise: When is Skilled Intuition Useful?

Decision-making in professional sports contexts is clearly complex and multi-faceted. Decisions, in subtly different contexts, will hold differing degrees of predictive validity. For example, selecting tactics for a specific game, prescribing team conditioning strategies and deciding on a player’s rehabilitation framework are clearly different decisions with distinct predictive validities. These decisions are taken in similar contexts, and often by the same collectives of practitioners. Yet, depending on the predictive validity of the specific phenomenon, the relevance of intuition gained through prior experience may vary widely. As an analogy, consider a meteorological example: Experienced weather forecasters are relatively good at predicting temperature and precipitation outcomes. This may lead forecasters to believe they are similarly effective at predicting other meteorological outcomes, such as hail. However, evidence suggests forecasters are ineffective at predicting hail [26].

Decision-makers in complex environments are faced with a spectrum of decisions ranging from highly predictive, where prior experience and informed intuition hold positive utility, to decisions taken in contexts holding zero-validity, within which experience and/or perceived expertise add little, or no, decision-making efficacy. Kahneman and Klein referred to this phenomenon as *fractionated expertise* [25]. The logical implication is that true forecasting and decision-making expertise is only feasible in a limited subset of complex contexts. Outside of these contexts, the complexity of the problems exceeds human computational capacity.

Paradoxically, however, experienced professionals are typically adept at constructing compelling narratives justifying the correctness of their forecasts [39]. Experts can, typically, eloquently explain why their predictions did, nearly did, or should have, come true [6]—even when such convictions are not substantiated. True expertise, subsequently, demands an ability to discern which phenomena provide observable and reliable patterns, adequately informing and educating intuitions, and which phenomena do not. This suggests that true expertise demands a capacity to distinguish between reliable and predictable challenges and those which are unreliable and inherently anomalous [13]. True expertise, accordingly, depends upon a capacity to recognise when intuition is useful, and when it is misleading [40].

### Traits and Characteristics of Good Decision-Makers

Following examination of data generated from forecasting tournaments, Tetlock characterised top forecasters as pragmatic experts who drew on many tools, sources and perspectives to gather as much information as possible before forming subsequent judgments [41]. These top performers shared core traits and cognitive styles, such as an eagerness to question beliefs; to investigate different perspectives; to be open-minded; to welcome opposing viewpoints; to think in probabilities and possibilities; and a willingness to readily admit when they were wrong, and quickly move on. In Tetlock’s words, these experts demonstrated "modest but real foresight, and the critical ingredient was the style of thinking" [5]. Mellers and colleagues illustrated that these forecasters had extensive domain-specific expertise and were characterised as *“better at inductive reasoning, pattern detection, cognitive flexibility, and open-mindedness*” [41].

Crucially, high performers viewed forecasting and decision-making not as an inevitable outcome of experience alone, but rather as a set of skills requiring deliberate practice, sustained effort, constant monitoring and continual accumulation and scrutiny of emerging evidence [29, 42]. As noted by Tetlock, the best forecasters exhibited characteristics indicative of intelligence, curiosity and knowledge-seeking behaviours that were reflective and analytical with a deep-seated need for cognition and understanding [5, 42].

## Optimising Professional Sports Decision-Making: Promoting Awareness and Education

The lessons emerging from the GJP suggest that specific personal traits and habits, if instilled or amplified in individual decision-makers, serve to enhance judgement and decision-making abilities [41]. A summary of these traits and habits is provided in Table 1.

**Table 1:** The key traits and habits of effective decision-makers

Traits and characteristics	Description
Analytical	Seek a diverse range of opinions, rather than looking for personal validation
Actively open-minded	Beliefs are treated as hypotheses to be tested, not treasures to be protected
Thoughtful updaters	When the facts change, beliefs are quickly updated
Growth mindset	Recognise decision-making as a skill demanding diligent and scrutinised reflection

Similarly, at a group and/or organisational level, judgement and decision-making performance can be improved by refining organisational cultures and processes and introducing training initiatives. In summarising the conclusions of the GJP, Mellers and colleagues recommended 3 broad interventional targets designed to accelerate learning and promote the evolution of enhanced collective decision-making processes [41] (see Table 2.)

**Table 2:** Good Judgement Project strategies to enhance effective decision-making

Strategy	What	How	Why	Example
Team It	Targeted training focussing on harnessing the power of MDTs	Training in collaboration and fostering core behaviours of MDTs	Mitigating common cognitive pitfalls such as groupthink and social loafing, etc.	Strategically designed processes promoting psychologically safe environments encouraging diverse opinions
Train It	Targeted education focussed on enhancing DM knowledge and awareness	Education raising awareness of cognitive biases and debiasing strategies	Mitigation of common cognitive pitfalls, such as overconfidence and belief perseverance	Avoiding unilateral decisions by fostering peer-group reflections and consultations
Track It	Systematic tracking of DM processes and development of metrics quantifying DM ‘success’	Continuously revaluating and updating forecasts and decisions	Maintain DM modifiability based on emerging information	Create processes for the review and consideration of emerging information

## Conclusion

The effectiveness of professional sports practitioners is, in large part, a product of the quality of their decisions. Yet culturally, within professional sport, as evidenced by the absence of decision-making training initiatives in coaching and/or sports science educational curriculums, we typically neglect this dimension of staff development. Although good forecasting and decision-making proficiency are clearly essential facets of good professional practice, sports science providers do not—either within formal sports science curriculums or discipline-specific certifications—receive any education on the basic principles of effective decision-making. Importantly, growing evidence clearly illustrates that forecasting and decision-making are malleable and trainable skills that, if appropriately targeted and practiced, can be efficiently and effectively improved.

Deeper understanding of the traits and characteristics of good forecasters and decision-making fosters more pragmatic decision-making cultures and processes and provides sporting clubs and organisations with opportunities to mitigate the most pervasive decision-making traps. Specifically, the evidence reviewed here suggests:

- Expertise is not a natural outcome of experience
- Experience alone will not enhance forecasting and decision-making abilities, and may promote misguided over-confidence
- Sporting organisations should appropriately educate staff, create open and trusting environments, promote collaboration and engineer appropriate decision-making processes

In closing, IARPA’s basic message to the intelligence community can be broadly summarised into 3 key take-home recommendations to enhance decision-making cultures:

- *Design* engaging professional cultures that harness the power of collectives and encourage diverse opinions, perspectives and collaborative teamwork, whilst simultaneously defusing and deflating ego threats, decision-making arrogance, over-confidence and groupthink
- *Educate* practitioners by providing initiatives promoting probabilistic thinking, statistical reasoning and awareness of cognitive biases (specifically targeting debiasing training strategies)
- *Evaluate* decision-making outcomes rigorously and provide real-time feedback so staff can score forecasts and thereby hold their theories accountable with meaningful metrics

Decision-making expertise is, ultimately, not a passively absorbed, inevitable outcome of experience. Decision-making expertise, instead, requires active and strategic engagement with, and post hoc review and evaluation of, learning opportunities. Promoting decision-making expertise within professional sports organisations demands that the traits and characteristics of good decision-makers are amplified through strategic education, appropriately engineered decision-making processes and actively challenged through targeted training initiatives.

## Data Availability

Not applicable.

## Abbreviations

IARPA: Intelligence Advanced Research Projects Activity
IC: Intelligence community
VUCA: Volatile, uncertain, complex, ambiguous
NDM: Naturalistic decision-making
GJP: Good Judgement Project
